# Advancements in Gel Science—A Special Issue in the Memory of Toyoichi Tanaka

**DOI:** 10.3390/gels5030033

**Published:** 2019-06-26

**Authors:** Masayuki Tokita

**Affiliations:** Professor Emeritus, Kyushu University, Fukuoka, Japan; northbear3.14@gmail.com

It is a great pleasure for us to present a collection of recent papers that were submitted to the special issue of Gels, Advancements in Gel Science—A Special Issue in Memory of Toyoichi Tanaka. The science of gel attracts much attention from many scientists who work in the fields of physics, chemistry, and biology. The famous chemist, P. J. Flory at Stanford University, has built the fundamentals of the gel science. His brilliant achievements are published in many journals and, finally, knowledge about the science of not only gel, but also the physical chemistry of polymers is summarized in his “Bible”, Principles of Polymer Chemistry, which was published in 1953 by Cornell University Press.

In 1977, the physicist Toyoich Tanaka at Massachusetts Institute of Technology achieved a great breakthrough in gel science, with the discovery of the critical phenomena and the volume phase transition of gels. After the discovery, Toyo, which was his nickname in the lab, cultivated the new era of gel science. His science is based on experimental study, though he also had deep insight into theoretical physics as well as biological physics. Therefore, the papers he published are excellent guides for researches who are working in the area of physics, chemistry, and biology, even today. Many scientists expected further expansion of Toyo’s gel science, however Toyo suddenly passed away on 20 May 2000 at the age of 54. His papers that are related to significant discoveries were selected from his publications, which expanded most areas of science, and were published in 2002 by Tokyo University Press as a collection of Toyo’s posthumous works, named From Gels to Life. 

The year 2017 was the 40^th^ anniversary for the discovery of the volume phase transition of gel. It is, therefore, a timely opportunity to summarize the recent advancements of gel science from this year. As a result, we, MDPI, planned to publish a special issue of Gels. In this special issue, fortunately, 14 papers on the recent advancements of gel science in the areas of physics, chemistry, and biological science are summarized. I believe that all papers published here are of interest scientifically and that they have a significant impact on each area of science. We thank all scientists who cooperated with our plan and submitted their recent research work on gels to this special issue. We hope that this special issue becomes a milestone for gel science for the next 10 years.

